# Contribution of community-based sociotherapy interventions for the psychological well-being of Rwandan youths born to genocide perpetrators and survivors: analysis of the stories telling of a sociotherapy approach

**DOI:** 10.1186/s40359-020-00471-9

**Published:** 2020-09-29

**Authors:** Emmanuel Biracyaza, Samuel Habimana

**Affiliations:** 1 District Manager of the Sociotherapy Programme, Prison Fellowship Rwanda (PFR), Embassy of the Kingdom of the Netherlands (EKN) project, Southern Province, P.O Box: 2098, Kigali, Rwanda; 2 Rwanda Resilience and Grounding Organization (RRGO), Kigali, Rwanda

**Keywords:** Sociotherapy, Youth, Transactional analysis, Survivor, Perpetrator, Hedonic well-being, Eudaimonic well-being, Narrative data, Field work

## Abstract

**Background:**

Psychological well-being (PWB) refers to inter- and intra-individual levels of positive functioning that include one’s relatedness with others and self-referent attitudes that include one’s sense of mastery and personal growth. PWB consists of hedonism and eudaimonia building on thoughts, feelings, and behaviors. Sociotherapy is a community-based health intervention that contributes to the promotion of PWB worldwide. Starting from an analysis of trauma transmitted from the perpetrators and survivors of the Rwandan genocide to their descendants, this article is aimed at exploring the contribution of therapeutic sessions to PWB among youth.

**Method:**

A qualitative study design based on shared testimonies was carried out. Field reports from the sociotherapists, written texts, and testimonies of changes presented in the conviviality meetings were analyzed using transactional analysis. We included 24 reflexive texts upon completion of 8 months of the therapeutic program.

**Results:**

Results indicated that before sociotherapy sessions, youth born to genocide survivors and perpetrators had psychosocial distress, including low self-esteem, hopelessness, anxiety, stigma, thoughts of revenge, shame, depression, and antisocial behaviors. Sociotherapy significantly contributed to the reduction of these psychosocial problems. Participating youth reported feeling safe, trusted, respected, and healthier than before the sociotherapy. This intervention created inner healing, social cohesion, alleviated their sufferings of trauma, restored their families and contributed to community resilience. Results revealed that youth developed PWB, helping them to what appeared to be psychosocial problems as being potentially healthy, enhancing self-acceptance, and respecting humanity. Youth also became the vector for the reconciliation and reconstruction of their humanity.

**Conclusion:**

Sociotherapy is a community health intervention that has an effective outcome on the personal well-being of of youth. This therapy impacted the individual, social, and familial resilience of youth who developed their capacity to regain and maintain health. The intervention restored their PWB, characterized by increased positive functioning specifically in the areas of autonomy, ecological mastery, individual growth, purpose in life, good relationships with others, and improved self-esteem.

## Background

Mental disorders remain an unfinished agenda worldwide [1] and are highly prevalent in low and middle income countries (LMICs) [2, 3]. This high occurrence is influenced by less prioritization for mental health services and insufficiency of mental health interventions [4]. It was found that more than 70% of the global mental health burden occurs in LMICs [5]. The American Psychological Association (APA) developed guidelines that play a crucial role in preventing mental health disorders by promoting a preventive perspective in psychological well-being (PWB). These preventive measures are important in reducing the risk factors for mental disorders, treating mental disorders, and decreasing the likelihood of developing negative mental health outcomes due to various factors, including traumatic events. These interventions also build strength in the individual, the family and society [6, 7].

Psychological health simultaneously refers to the absence of the crippling elements of the human experience various mental disorders that include the depressive disorders, psychotic disorders, personality disorders, psychosomatic disorders, anxiety disorders and phobia [8]. Such other scholars discovered that the PWB consists of positive relationships with others, engagement, positive emotions, meaning, healthy relationships, self-actualization, personal and environmental mastery, autonomy, a feeling of purpose and meaning in life, and personal growth and development [8–12]. The first of these, positive relationships with others, refers to the extent to which people experience positive emotions and feelings of happiness in their interactions with other people. Sometimes this aspect of psychological well-being is referred to as subjective well-being [13]. PWB as a fundamental state of people, in their daily functioning of the individuals and maintain the community wellbeing being and include the hedonic and eudaimonia components which are the components including logical thinking or spirituality (such as meditation, mindfulness practices), emotional equilibrium (such as generosity, empathy, and compassion) and evaluative satisfaction with the respect to various domains such as engagement, social cohesion, meaning and purpose, mastery or achievement and mental illness [8, 14, 15]. This psychological well-being is characterized by the reduction of mental health burdens. However, many mentally-ill people remain untreated, although many effective psychological treatments and interventions are available [15].

PWB refers to inter- and intra-individual levels of positive functioning that can include one’s relationships with others and self-referent attitudes that include one’s sense of mastery and personal growth. Subjective well-being reflects dimensions of affect judgments of life satisfaction [14, 16]. There are two types of PWB, hedonism and eudaimonia, that encompass three components that include logical thinking, positive feelings or emotions, and positive behaviors [17]. The concept of “hedonic” well-being is normally used to refer to an individual’s subjective feelings of happiness and contentment; hedonic PWB possesses both affective and cognitive components. The affective component consists of high positive and low negative affect such as the presence of positive mood. Regarding cognitive component, it includes global life satisfaction. It is proposed that an individual experiences happiness when positive affect and life satisfaction are both at high levels [6]. Most researchers confirm that both hedonia and eudaimonia are fundamental for individuals to flourish, build strong mental health, and restore equilibrium. While these two terms, hedonia and eudaimonia, are not opposites, they also are not mutually exclusive, and should be seen as complementary psychological functions [18].

Through this definition of PWB, any negative symptoms are managed, with the individual developing positive emotions and normal behaviors that make up healthy relationships, meaning, environmental mastery, engagement, and self-actualization. Earlier we stated that PWB is characterized by self-acceptance, life satisfaction, life purpose, personal growth, environmental mastery, autonomy and positive relationships with others [19]. Psychological well-being is above and beyond the absence of psychological distress or illness, and is considered part of a broader spectrum of constructs than the traditional conception happiness [20]. PWB includes the absence of schizophrenia and other psychotic disorders, personality disorders and mental retardation, mood disorders, anxiety disorders, dissociative disorders, adjustment disorders, factitious disorders, somatoform disorders, sexual and gender disorders, sleeping and eating disorders, impulse control disorders and mental disorders due to general medical conditions, substance related disorders, delirium, dementia, amnesia and other cognitive disorders [21]. An individual suffering from mental disorders may not be able to experience PWB. However, the absence of those disorders does not guarantee psychological flourishing. Since society traditionally supports mental illness within its healthcare system, it belongs in the health domain and thus will not be a focus of this chapter. Only some of the interventions described below can both alleviate mental illness and improve positive psychological functioning [22].

In PWB, individuals with psychological disorders also fight for developing mental health by healing emotionally and behaviorally painful inner conflicts. Through these ways of reconstructing PWB and bringing the central nervous system (CNS) into equilibrium, individuals can stop resisting, controlling, censoring, judging, and interpreting their uncomfortable feelings and emotions [20]. Integrating themselves with their feelings, and not standing outside them or attempting to control them, restores natural wholeness of being, which removes the abnormal and psychologically unhealthy inner division that is the basic source of innermost conflicts? That natural state of undivided wholeness and nonjudgmental, unconditional acceptance of our feelings is the basis of psychological healing [23]. Prior studies confirmed that healing ourselves of emotionally painful inner conflicts also involves releasing our energy and conscious attention from blocked or obscured by the imposition of our own self-definitions, self-evaluations, and rigid social roles [20]. Other studies indicated that for self-healing to occur, the individual must fully embrace emotional pain and relationships with an open, nonjudgmental, nonpartisan, compassionate, and warmly loving heart [23]. As stated previously, studies have indicated that the theoretical model of PWB encompasses 6 distinct dimensions of wellness, including autonomy, environmental mastery, personal growth, positive relations with others, purpose in life, and self-acceptance [24, 25]. Although these earlier studies did not focus on community-based interventions such as sociotherapy, still other studies parsed well-being according to global questions about overall life satisfaction and domain-specific questions about work, income, social relationships, and neighborhood [24, 26]. Recent studies have indicated that PWB that is composed of life satisfaction and happiness is characterized by two concepts: meaning of life and self-determination [27].

Rwandan youth have been affected by the domestic violence that are still affecting most of the population of the first and the second generations [28]. Studies showed that the 1994 genocide against the Tutsi has been a vector for the transmission of domestic violence in Rwanda [29], and in the aftermath of the genocide, economic, environmental, mental and physical health problems, along with academic problems among children, have been the main factors contributing to domestic violence [30]. The genocide continues to have a negative impact on conjugal relationships, in which the prevalence of trauma associated with it is still high, and traumatic marital conflict among the second generation remains a public health issue [30]. It was found that posttraumatic stress disorder (PTSD) currently stands at 26.1% of the population, and has an impact on family life [31, 32]. Research in Rwanda highlighted the presence of heterotypic continuity, a term that refers to the transmission of violence or other maladapted behaviors from one generation to the next [33]. This has a manifold impact on both individuals and society, such as mistaken expectations of marriage, separation, divorce, depression, inadequate preparation, perceived failure to take on tasks, poor communication, and unilateral decision-making within Rwandan families [34].

The direct pathways of intergenerational transmission concern the manner in which the genocide and other violent acts, as well as their aftermath and related events, are reflected upon, reconstructed, and either kept secret or explicitly communicated to the younger generation. The indirect pathways of intergenerational transmission concern the manner in which the genocide and its aftermath affect the second generation’s socio-ecological environment, and thus, the children’s social environment. The effects can be felt on parenting, family structures, family functioning, the family’s socio-economic status [35]. It has been postulated that the family is the prime conduit of memory for trauma and trauma-related PTSD [36]. Whether or not parents disclose their traumatic experiences (e.g., genocide) to their offspring and descendants, they transmit their bodily and affective connections to these experiences. This raises the question of whether the next generation has a tendency to exhibit hatred, fear, revenge, anger, and violence toward former adversaries and their descendants, or whether they have a propensity to go beyond past divisions in society [28, 37]. The path taken by the parents depends not only on family dynamics but also on how the memory of trauma is passed on in the community and wider society, and the ways that the different levels of memory transmission influence each other. Previous findings indicate that children are deeply affected by what their parents pass on to them in terms of memories, behaviors, attitudes and emotions, but in different ways that partly depend on their parents’ sociohistorical background [38]. Additionally, it was indicated that intergenerational legacies of genocide are a risk factor for the internalization of feelings of genesis of divisionism (sectarianism and ethnic division) that are vague and sweeping, and have been used to silence legitimate dissent. These harmful acts ideology and sectarianism resulted in the 1994 terrible genocide against the Tutsi. The genocide and its aftermath created the genocide ideology among the second generation and this has been resolved though diverse efforts of the government, national and international contributors with the goal to bring the unity, reconciliation, nationalism, patriotism, dignity, and rehumanisation [33, 39, 40]. A high prevalence of PTSD has been documented in Rwanda since 1994, the year in which the genocide against the Tutsi took place [41]. Concerning the mental health problems in Rwandans, PTSD among the genocide survivors and the transmission of trauma to their descendants and other neighbors was reported [30, 42]. In addition, other studies documented the detrimental effects of the genocide on families living where the generational trauma actually occurred. Research conducted between 1994 and 2019 revealed an increase in domestic violence and trauma, and showed that it has a negative impact on (genocide survivors’ and genocide perpetrators) living conditions, socio-economic development, and their families [43]. These impacts are predominantly characterized by the experience of trauma and its effects among people who were directly and indirectly affected. The destruction of the social fabric intra-conflicts in Rwandan remains a problematic issue nationwide.

Psychological problems among Rwandan youth continue to increase in the aftermath of the genocide carried out against the Tutsi. Youth born both to genocide perpetrators and survivors were intensely affected due to the traumatic experiences they faced and/or the touchable histories (personal histories) of their parents and relatives in the form of transgenerational trauma [31]. Very little is known about how sociotherapy reconstructs PWB (hedonic and eudaimonic) among Rwanda youth in the aftermath of genocide against the Tutsi in Rwanda. Accordingly, this study was carried out using dialogues shared in sociotherapy sessions and testimonies given by youth during conviviality meetings, which they attended after completing six phases of sociotherapy sessions. The study was designed to assess the contribution of community-based sociotherapy to PWB of Rwandan youths born to genocide survivors and perpetrators.

## Methods

### Study design

The study design used was an exploratory qualitative method based on a case study approach, represented by participant responses given in the sociotherapy sessions and conviviality meetings. Qualitative research is a systematic scientific inquiry which seeks to build a holistic, largely narrative, description to inform the researcher’s understanding of a social or cultural phenomenon [44]. The case study was used for describing an entity that forms psychosocial well-being among youths assigned to the community-based sociotherapy intervention in the southern province of Rwanda. Grounded theory, in which interviews with experts in a specific area are conducted for the purpose of developing a theory grounded in data from the field, was used as the basis for data collection and analysis for the qualitative components of this study.

### Study setting and participants

This study included youth from the Nyamagabe, Nyanza, and Muhanga districts located in the southern province of Rwanda where the sociotherapy intervention was implemented. The project included youth who were recruited from these districts, the majority of whom were born to genocide survivors and perpetrators. This study included youth aged 20 to 30 years who had been affected by transgenerational trauma related to the effects of the genocide and its aftermath. The study excluded participants (a) who were adolescents during the 1994 genocide against the Tutsi, or (b) who were not descendants of families affected by the genocide, either perpetrators or survivors. All youth were participants of the Prison Fellowship Rwanda program sponsored by the Embassy of the Kingdom of the Netherlands (EKN). Prison Fellowship Rwanda is a local non-governmental organization (NGO) that collaborated with Community-Based Sociotherapy (CBS) Rwanda, and the EAR Byumba Diocese (the Anglican Church in Rwanda) to implement the sociotherapeutic intervention in Rwanda.

### Data collection and materials

The data were collected by the district manager and sociotherapists who facilitated each group session. The field coordinator and field researcher, also involved in the daily activities related to the group sessions, also participated in data collection. The sociotherapists gathered the reports and submitted them to the district manager, who monitored group sessions in the southern province. Data were also collected from the sociotherapists’ reports which arose from the group sessions. In the group sessions, participants shared their past experiences and histories. The district manager also monitored group sessions to gather information about participants’ health issues and the phases of the sociotherapeutic healing process. During documentation of the intervention, the following questions were addressed. The reports produced for the current study covered the entire 15-week intervention. One report detailed the first 4 phases (11 weeks) of the program, while a second report covering the last 2 phases (4 weeks) assessed the effectiveness of the sociotherapy intervention.

Format and issues covered in the first report included (a) Principles used in the session;, (b) Health problems discussed in the last sociotherapy session; (c) Current efficacy and effect of interventions discussed in the last session; (d) Any new health problems brought up in the current group session; (e) Any problems discussed in the day’s session; (f) ideas/interventions about problems discussed in the day’s session, Health problems shared in the session and ideas/approaches to put into practice; (g) Problems raised in the session but not discussed; and (h) Supports and aids needed from PFR staff in order to provide effective and efficient group facilitation?

In the second report, we used the format from the fifth and sixth phases, which took place in the 11th week. The questions were (a) Questions about and principles of confidentiality used in the therapeutic session; (b) Health problems discussed in the last session; (c) New life orientations taken in the last session or the current session, or memories of sociotherapy participants raised in the memory phase (if a health problem in the group session, it may be discussed; (d) Contribution of the intervention to the participant’s new life orientation, to treating and preventing the psychological effects of traumatic experiences encountered by the sociotherapy participants (if possible, the other paper to write the testimony of change may be used); (e) Health problems brought up in the group session and a detailed description of the intervention provided by the group participants; and (f) Supports and aids needed from PFR staff in order for sociotherapists to provide effective and efficient group facilitation.

One tool used by sociotherapists was a reporting template that contained sample questions for each phase of the therapeutic sessions. Daily reports and testimonies were shared with at the end of each session and during the conviviality meetings. The researcher collected the life histories and statements of the particinapts as well as their testimonies of changes after joining sociotherapeutic sessions. The reports were also adapted to preserve confidentiality between the implementers, sociotherapists, and participants. The report format did not contain the names of participants who shared information. Another tool documented the steps followed in the group sessions, such as sharing news from the last week, evaluating changes from the last session, introducing the next phase, discussing traumatic events that affected the participant, presenting problem-solving ideas from the group participants, closing the session, and reminding participants about the next session.

### Data analysis

The materials analyzed were texts written as reflexive opinions and answers gathered during the group sessions and conviviality meetings. The reports sociotherapists submitted to the district manager were also analyzed. These data were accurate and collected by the trained sociotherapists, District Manager, field researcher, or field coordinators. In the current study, there was no need to categorize participants’ experiences into categories for quantifiable measurement [45]. The materials used for analysis included reflexive reports written by the youth or the sociotherapists involved and participating in the intervention. The current study presents results of the transactional analysis of 24 texts written upon completion of two rounds of an 8 month therapeutic program. The youth recruited were involved in the first two rounds of the program implemented from April 2019 to December 2019.

The following methods were used to analyze data collected from these two rounds of the program:

- Reading and understanding the texts reported, and selecting some texts for more detailed transactional analysis;
- Selecting texts that reflect the most extensive range of experience and points of view;
- Reviewing the testimonies of change, understanding them based on the participant’s status before sociotherapy session, how the group sessions contributed to his psychological health, and the new decisions or new life orientation taken.
- Analyzing the texts the reports and testimonies of change individually to extract meanings and outcomes of the sociotherapeutic intervention.

The methodological basis of the transactional approach (TA) was based on findings presented in reports compiled by the program’s district manager. Transactional analysis (TA) was developed by Eric Berne, inspired by Sigmund Freud’s theories of personality [46, 47]. TA is a psychoanalytic theory and therapeutic method in which social transactions are analyzed to determine the subject’s ego as a basis for understanding behaviors [46]. The goal of transactional analysis, a form of modern psychology which examines an individual’s relationships and interactions, is to reinforce the idea that each individual is valuable and has the capacity for positive change and personal growth through therapy or an intervention [47]. The main principle of our hypothesis is that sociotherapy is an effective therapy that contributes to the psychological well-being of Rwandan youth born to the perpetrators and survivors of the 1994 genocide against the Tutsi.

### Understanding the sociotherapeutic approach

Sociotherapy is an interventional approach that has been found to contribute to the social cohesion of individuals from the same neighborhood or group [39]**.** This approach is characterized by a group of 10 to 15 participants sitting in a circle, as a forum for talking about their experienced health stressors and the effects on their psychosocial health. This intervention aims to restore psychosocial health of the participants and community and it contributes to economic welfare [48]. Participants also talk about events related to trauma, sexual or gender-based violence, war, genocide, and other public health concerns, and the destruction of trust and safety within their communities, as being akin to “life without humanity” [49]. To facilitate a sense of redress for people in Rwanda, an approach was needed to address psychological factors operating at both the individual and community levels. Therefore, we set out to analyze the PTSD associated with the 1994 Rwandan genocide against the Tutsi as one of the root causes of domestic violence in Rwanda, with some research postulating that domestic violence has been a vector for the transmission of trauma to children in the aftermath of the genocide [33]. Sociotherapy is applied in the group participants’ community within which they can share their past experiences and the effects on their mental and social functioning. Through these discussions, organized as a series of phased sessions, the participants expressed the benefits they derived from the dialogues [50].

This approach is based on six phases focusing on different issues, including safety, trust, care, respect, new life orientation, and memories. The first four phases are spent discussing the psychosocial issues affecting the individuals and the community, and the two last phases are principally used to evaluate the outcomes of the group sessions. The order in which safety, trust, and care are developed is of major social importance, because learning to delay gratification of urgent emotional needs makes it possible for participants to discuss difficult subjects at a later, safer, time. For this reason, processing unpleasant emotions is done in the sixth phase of group development [40, 48].

Each phase of sociotherapy is designed to contribute to the participants’ psychological well-being. In the “safety” phase, the goal is to create an atmosphere in which each participant feels safe in group discussions. Because loss of trust in individuals and institutions is one of the effects of exposure to trauma, in the second phase, “trust,” participants come together to restore the trust that was lost in each participant’s history, whether perpetrator or survivor. Rebuilding trust in the space between cognitive and social-emotional attention, and caring for the individual within the group, is essential to bringing social cohesion and psychological healing to each participant in the group [48]. The third phase, “care,” focused on participants who had not been cared for, lost self-care, or for whom caring for others was difficult due to the harmful events they had experienced. In this stage, each participant develops sympathy for individual group members, and the group dynamics come alive, with the group now acting as a carrier of social events [51]. In the fourth phase, “respect,” participants express how they were not respected and were discouraged from respecting others due to the different factors that psychosocially affected them. It is essential that respect is reconstructed in the group, and that all the individuals in the group reshape their humanity and dignity, and strengthen their rights as human beings [52]. In the fifth phase, “new life orientation” (former rules), participants compare their behaviors, emotions, and feelings before group sessions, what participating in the group contributed to their lives, and their new focus on becoming healthier. In this phase, the participants discuss the outcomes of the sociotherapy sessions. In the last phase, “memories,” participants remember their past traumas, and re-experience the emotions that disrupted the homeostasis of their lives. After remembering the good and the bad events, the participants reconstruct themselves [48, 51].

### Ethical considerations

In the sociotherapeutic intervention, all information shared by the participants was kept confidential. Participation was understood to be voluntary and that each participant had the right to withdraw at any time. Only the district manager and two sociotherapists from each group had access to the demographic information collected, which they monitored to assess the program’s impact. The sessions took place over 15 weeks, divided into six sociotherapeutic phases, with each weekly session lasting 3 h. Regarding confidentiality, the participants themselves created the rules at the first session, which were respected in their journey of intervention. Of these, privacy, active listening, and confidentiality formed the foundation for each session. As the protection of the participants is respected and it is included in the project, the ethical approval from the institutional review board was not searched for; this means that participants essentially created their own rules, that IRB approval was not needed. The rules were verbally repeated before each session, and based on the agreement between the participants, sociotherapists and District Manager, the participants shared the changes they had experienced as a result of the sessions. In the first session of the intervention, the participants created their own code of group conduct to be used in the sessions, specifically the agreement regarding privacy, confidentiality, and active listening. These rules come to complement the seven recommended principles in sociotherapy intervention such as democracy, responsibility, interest, participation, equality, learning by doing as well as here and now are essentially stated and unforgettable rules in the session [40]. Through these principles, participants saw they were expected to contribute equally to the process, establish and actively engage in group dialogue, make decisions together, and work towards mutual cooperation [53]. Therefore, we used the prior approval for conducting the current study.

## Results

At the end of the sociotherapy sessions, youth were found to have benefited from these interventions, reflecting various themes which expressed the effectiveness of sociotherapy on the participants’ psychological well-being.

### Psychological healing

The problems brought up in group sessions included mood disorders such as anxiety and depression, as well as sleep disturbances, stigma, and trauma. Results from the participants’ testimonies indicated that participants healed their psychosocial problems, and saw sociotherapy as medicine for youth born to both perpetrators and survivors of the genocide.

*“Sociotherapy is the medicine that healed me and restored my dignity and humanity.”*

(Pauline, female aged around 27 years)

*“After joining the group, I was able to get back on the right track of my life and overcome the problems I was facing in my daily life. I learned that nothing is impossible. I hope my future life is better. Therapeutic group helped me to realize that I am capable of doing something to improve and develop my life. My family had forbidden me to bring pigs in the house but I realized that the problem was that I was asking for their permission in a wrong manner. Now they permitted me to raise pigs.”*

(Clemence, female aged around 23 years)

Youth indicated that before sociotherapy sessions, they were afraid of neighbors from different ethnic groups. Youth descended from genocide perpetrators were afraid of those descended from genocide survivors and vice versa. Youth from survivors thought that youth whose families were genocide perpetrators were also murderers. One youth, whose parent is in prison for genocide crimes, said they lacked parental care and through sociotherapy, they felt cared for:

*“Through sociotherapy, I was treated like my second parents.”*

(Julius, male in his 23s)

Participants whose parents were genocide survivors said they and their own children were negatively affected. With group therapy, youth become agents of change for their parents, helping to improve communication in the family. They shared changes in their parents’ abnormal behaviors and irrational thoughts:

*“Usually a parent changes a child's mind and behavior but I have found that we young people also are able and powerful to change parents about the history of the genocide and its consequences.”*

(Ericus, male aged around 26 years)

“*I talked to my fellows in the group and they gave me different constructive thoughts that healed me. Sociotherapy helped me and my wounded heart was healed. Then I was able to talk to my mother about harmful things she does for me and she promised that it will not happen again. Nowadays, when she wants to ask me something, we sit together and talk (about) it. The sessions allowed me to find a way of communicating with my mother. Grudge for people who killed my family members was healed. The therapy helped me to heal my traumatized mother.*”

(José, female aged around 25 years)

### Growth, development and personality changes

The findings demonstrated that the psychological well-being of youths was restored through the dimensions of autonomy, environmental mastery, personal growth, positive relations with others, purpose in life, and self-acceptance. Sociotherapy contributed to the reconstruction of the community’s mental health and restored family cohesion that had been destroyed by the effects of the genocide and its aftermath. This approach contributes to personality change, development and maturity in the participants:

*“I used to be angry because of the anger about who killed my family and this continues at the beginning of the session. At the fifth phase of new life orientation my heart became restored.”*

(Emmanuel, male in his 20s)

“*Phase of memories helped me to know that genocide occurred and caused me to lack paternal care. … it also helped me to become able to develop myself and develop confidence and empathy.*”

(Wolfe, female in her 25s)

*“Sociotherapy therapy helped me to have positive feeling, and cope with abnormal behaviors and thoughts such as stigma and shame. Using the ideas learned from the group, I also tried to change the mindset of my mother and then our family no longer has conflicts as it was before. I became social being and have known that I am human being who has respect and vision”.*

(Susanne, female aged around 27 years)

### Social cohesion and family relationships restored

In the sociotherapy sessions, participants developed eudaimonic states to help them regain the happiness and contentment lost due to their past experiences. The lives of youth born after the genocide were characterized by long-term dysfunctional relationships, often imbued with rampant violence, creating significant obstacles to the therapeutic work in which they were engaged. These relationships had devastating effects on their lives, families and school performance before the sociotherapy sessions. In the group sessions, these effects were controlled and the process of rebuilding PWB began. One participant attested:

*“I had no friends because all the families hated me and my mother. Sociotherapy built my friendship starting from group participants who care(d for) me.”*

(Nathanaël, male aged around 28 years)

*“I didn’t know I hated others and was unable to sit with murderers and their descendants, but since I have joined sociotherapy I had found that murder is a human being who needed value and dignity.”*

(Chanto, female in her 20s)

In the six phases of the program, youth approached the intervention as a form of medication they could use to rebuild their mental health and social resilience. Their healing was also impacted by the effective dialogues shared by each participant, each of whom respected the rules that contributed to the sustainability and efficiency of the sociotherapy approach. It was found that the ability to be part of a team, and share and learn from each participant’s individual histories was the key to the process. The sociotherapy participants built relationships, learned to be together, work together, solve problems and cope with difficulties arising from the effects of genocide and its aftermath, which had been transmitted from their parents and other relatives.

### Sexual reproductive health problems

Young adulthood is the period for exploring and developing aspects of personality and behavior, often influenced by exposure to a variety of attitudes and ways of life that can impact their health*.* The sociotherapy participants developed mental well-being through their participation in the sessions held in their communities. Through this community health intervention, participants attested to improved self-control and competence. One young woman testified that the sessions contributed many psychological and social benefits, helping to restore lost trust and cope with feelings of anger, loneliness, and shame at having unwanted pregnancies. The shame, loss of responsibility, fear, and stigma did not allow her to attend the policies of the government or community activities. However, the trust phase of the program helped her to overcome the irrational thoughts related to the loss of trust, empowering her to attend the policies, and control her fear. She began helping others in the community who had not attended the sociotherapy sessions, and hopes the program will be implemented in a large area due to its positive contribution to health.

A participant said:

*“After providing early pregnancy, domestic violence started occurring in my family. Since I shared it to the group, my colleagues gave me constructive thoughts to implement. These helped me to become resilient and reconcile with my relatives and parents. I learned that Rwandans are unique … .I have the vision for changing other(s) in my community.”*

(Angelus, female in her 25s)

### Confidence and involvement in the community actions

Participants shared that before the sociotherapy sessions, they had suffered from various psychological problems, including disorganized behaviors and negative feelings caused by the trauma transmitted to them by their parents. Through the sessions, the participants indicated that they developed psychological and social well-being through self-control, self-acceptance, and personal growth. This intervention was found to have helped participants express themselves in their community. One participant attested:

“*From the third session, they started to respect me and give a time for sharing (with) the group my psychological problems. They gave me a constructive thought and made me social. Sociotherapy reconstructed my dignity and helped me to have hope for the future. As they no longer (avoid) me in the group but help (ed) me, this helped me to have again the trust. The more I attended well and shared my perspectives in the group, they coped with fear and then made me stronger. I wish to return to school in senior (year) five. I plan to (have a) relationship with my family and work hard for my vision and hope.*”

(Angelus, male aged around 25 years)

*“The group sessions helped me to understood and overcome the consequences of my parents whose different ethnic groups. Their conflicts caused me to my dropping out school. Now I am resilient.”*

(Susanne, female aged around 27 years)

### Reconciliation and forgiveness

The youth participants tended to give long-winded backstories, putting their actions in perspective within the greater social context, and describing how they benefited from the sociotherapy sessions. Youth indicated that they contributed to the process of reconciliation by participating in various activities, mostly in their families and communities. Among the activities related to reconciliation, participants helped their parents to forgive genocide perpetrators, request forgiveness from genocide survivors, pay restitution for property destroyed in the genocide, foster social cohesion, and reconstruct family affected by the genocide. Youth also contributed greatly to building social cohesion and promoting socio-cultural values. Said one participant born to a genocide survivor*:*

*“Sociotherapy helped me to cope with (feelings of) revenge to the families that killed my family. Then, I also coped with irrational thoughts of not getting married the descendant from the genocide perpetrators.”*

(Ericus, male in his 26s)

Through forgiveness, participants indicated that they benefited tremendously when they chose to forgive, a process that helped free them from the harmful past and allow them to realize their potential. This forgiveness also helps overcome limiting beliefs, thoughts, and attitudes. The forgiveness found in the process of sociotherapy was the crucial step that released participants’ mental and emotional energies, which they can now apply to creating better lives. A descendant of genocide survivors testified:

*“Group sessions helped me to have forgiveness for the perpetrators and their descendants. I did not believe that my child was married to a family member who committed genocide, but now I would allow it because I have forgiven them and found that they are just like everyone else.”*

(Lea, female aged around 27 years)

In terms of restoration, the former prisoners who participated in the genocide were influenced by their descendants who took part in sociotherapy. These individuals then decided to take responsibility for their actions and pay symbolic reparations to their victims in the form of apologies. The youth descendants of survivors took the first step to forgive each other, after realizing that they did not participate in the genocide, and recognizing the effects of the genocide on their psychological health. A descendant from a genocide former prisoner testified:

“*When I joined the team and met my fellows who had bigger problems than mine, they talked to me and I was able to heal them. Also, I learned the severity of my father’s crime of genocide. I decided to convince him to ask for forgiveness to the family that imprisoned him due to his crime. I also encouraged him to pay back the properties he destroyed during genocide.*”

(Pacis, male in his 25years)

Another participant said:

“*Sociotherapy sessions helped me to have again trust and become psychologically reconstructed because I found that there are other people like me who have faced difficult problems. As my father will return in our family in two years later from the prison, I am feeling well.*”

(Alice, female in her 24s)

Another participant testified:

*“They gave me constructive opinions. I coped with revenging plans on behalf of my family. I accepted that my father did genocide. I decided to request for forgiveness and start reconciliation process to the genocide survivors. The Tutsi I called enemies are my beloved friends and we have sharing the goods. They keep my secret and we invite each other for sharing the goods. Sociotherapy helped me to take the new orientation of life without genocide ideologies. It restored my PWB.*”

(Clara, female in her 27s)

After understanding the effect of their histories and on both their families and communities, youth born to genocide perpetrators who participated in the sociotherapy sessions encouraged their parents to ask forgiveness and reconcile with genocide survivors. One descendant of a family survivor testified:

“*I decided to convince my father to forgive those who murdered our family. Now I visit the family, which my father told me that they killed our family and they treat me well. I even talk and play with their children.*”

(Emmanuel, male in his 25s)

*“But when joining sociotherapy program, I talked to my fellows and they healed me. Now I have forgiven people who committed genocide crimes and I learned that all Hutus are not bad because some of them did hide my parents and they survived. I am committed to interacting with those people and their descendants. I will teach my parents to forgive the perpetrators and those who can’t afford to pay back our properties, will not be asked to pay.*”

(Francis, male in his 25s)

### Healing transgenerational trauma and social isolation

The youngsters born after genocide grew up in a culture of silence and trauma, developing psychological disorders transmitted by family members who had participated in or survived the genocide. They also had negative feelings and moods that included anxiety, sadness, loss of trust, depression, loneliness, and guilt. In their daily lives, they experienced loss of concentration, poor life satisfaction, and loss of interest. During the sessions, participants reported developing an increased positive mood, improved life satisfaction, interest, and hope. A youth born to a genocide survivor shared with other participants this testimony of change:

“*To come in the sociotherapy group sessions permitted me to be open and shared (with) the participants from my community about my sufferings and hate. They really provided to me the effective ideas that I found that I was the source of my problem. After being given the effective ideas, I reconciled with the victim of my sin. I was forgiven by them and there is the strong reconciliation. My children currently go in the households of the neighbors without any problem. Sociotherapy became my restorative and relaxation space. I appreciated and was mostly helped by the rule of secret/confidentiality, democracy and talking freely (about) my problem. I thank that I was recruited for participating in this effective intervention. If our group had the old people (adults), I would not attend it well or share my problem freely.*”

(Philipo, male aged around 26 years)

Results showed that in the trust phase of the sessions, participants began to change their behavior and then started the reconciliation process. A participant testified:

“*Since I have been coming in sociotherapy group sessions during 15 weeks, I psychosocially became restored. I found that there are others who have similar problems to mine, especially youth whose parents committed genocide. The ideas from the group session in new orientation phase helped to decide not revenge and getting married to anyone without ethnic discrimination*.”

(Peter, male in his 20s)

### Self-identification

In the group sessions, almost all participants shared information about health problems. These included lacking parental care, whether because their parents had been killed in the genocide, were imprisoned due to their actions in the genocide, were former prisoners, or were genocide survivors suffering trauma. There was also trauma associated with domestic violence occurring in their families, dropping out of school due to insufficient resources, posttraumatic disorders, depressive disorders, sexual and reproductive health problems, anxiety disorders, thoughts of revenge, and feelings of shame related to the crimes committed by their parents. Each participant who shared a problem was provided with ideas and suggestions by other group members about how to overcome the effects of these past experiences, in the process becoming more resilient, self-confident and self-evident. The participant testified:

“*The group sessions helped me to reconstruct the relationship between my mother and grandfather that was destroyed in and after genocide when grandfather failed hiding us against the murders. Now there is the unity and reconciliation between them because of sociotherapy. I no longer have anger. I am also a social being and (have) no fear*.”

(Obrey, male aged around 24s)

### Decision making, responsibility and self-identification

Youth who attended the sociotherapy sessions saw the results of their efforts to become decisive and responsible. The family member of a genocide perpetrator testified:

“*When I joined the group, my fellows helped me with advice, which helped to rebuild my relationship with my family. Today, I am (on) good terms with my mother to the extent that we agree on things and the same applies to the rest of the family members. When I meet with them, we sit down and talk about our life then advise each other. Before (the) sociotherapy program, I didn’t want to do things like poultry or animal rearing but now I have a cow, which my mother gave me for free. Now when I meet my mother, we have food and drinks together, something that was impossible before.*”

(Patience, female in her 26s)

Youth descended from genocide survivors testified that after healing trauma characterized by anxiety and the inability to cope with certain aspects of daily life, they started working toward sustainable socio-economic development:

“*I have no longer extreme fear, depression, and anxiety. I share with my colleagues including Hutu and Tutsi. I thank sociotherapy because all the distresses I had before the therapeutic sessions were coped with. I am working for getting the socio-economic development. I advise my family [genocide survivors] to forgive the genocide perpetrators. I advise my family to reconcile with the genocide perpetrator. I want to rebuilt the country with no discrimination and hatred.*”

(Clara, female in her 27s)

### Sexual reproductive health problems and addiction to alcohol and drug

Our results indicated that many young people were not interested in working when they could spend the day in cinema houses, using alcohol and drugs, or having unplanned or unwanted pregnancies due to trauma and other problems related to genocide. Avoidance and hatred of returning to their environment of origin was also observed among youth before sociotherapy, but after the sessions, participants testified to changes in their thinking, feelings, and behaviors. Some returned to their family homes, became more responsible in their reproductive decision making, and saw improvements in their sleeping patterns. One of the young ladies who participated in the therapeutic sessions testified:

“*Through the group therapy, I realized how I was not respected, and I did not care myself nor children … was it my first time talking about what I passed through during the genocide! Today, I sleep well without using drugs or alcohol.”*

(Lea, female in her 20s)

Another youth born to the perpetrator who became prisoner expressed:

*“I started to heal and my heart was relieved off the burden. I actually learned that I was the cause for the constant conflicts with my father yet I used to put all the blame on him. My groupmates convinced me that I should change my behaviors and now my family is in a good relationship. I took initiative and encouraged my father to pay back others’ properties he destroyed during the 1994 Genocide. I even promised to help him. Overall, I am happy that in sociotherapy, we are united and determined to keep working together.”*

(Aaron, male in his 25)

### Psychological emotions and feelings

Most of the descendants of former prisoners and survivors developed many negative emotions and feelings that affected their hedonia and eudaimonia. They constantly lacked enjoyment and feeling of humanization. They often developed feelings of unhappiness, sadness, anxiety, and anger, which needed psychological interventions to achieve better health. Through the sociotherapy sessions, participants testified that their negative emotions and feelings were better controlled. One participant said:

“*Through the sociotherapeutic sessions, I have no longer (feel) shame and anger but got healthier thoughts to live in harmony with the genocide survivor and no longer become afraid of being with their descendants.”*

(Noella, female in her 26s)

The descendants of perpetrators and survivors reported that the group dialogues helped build them up and become social beings. One participant said:

“*Before joining the group, I didn’t want to share about my life with anyone. Only in the group I tried at trust phase. Since my father has been prisoner, my heart was full of scars. I always felt lonely and hated myself. Sociotherapy discussions taught me that one can need help from someone else. It helped me to stop hating myself as I shared with the group my problem. They gave me constructive thoughts.*”

(Pauline, female aged around 27 years)

A youth born to a genocide perpetrator said:

“*When I joined the group, I learned that some of my colleagues had bigger issues and other(s) had smaller than mine. Group session allowed me to heal and regain hope. I realized that I am capable and I can achieve something in my life. I also took the initiative and encouraged my father to reunite with people whose properties he seized in the 1994 genocide and also pay them back. In fact, I helped him to start paying back. I work hard to make this happen because I know that if they came for the payments, they would auction our properties, including my own. Sociotherapy helped me to realize that I am capable of more great things than I imagined before. My heart was restored.”*

(Paul, male in his 23s)

A descendant of a genocide survivor said:

*“When I found sociotherapy program, I got the medicine I needed. Now I am calm and no longer ask my father about his history because I met other youth in the group who experienced more painful tragedies. I am fine now and I even visit the family of the people who killed my relatives and they too visit us.”*

(Paulin, male in his 25s)

## Discussion

Our results revealed that youth born to both genocide survivors and perpetrators experienced negative psychological effects. Among youth descended from genocide perpetrators, some whose parents were prisoners or ex-prisoners experienced psychological and social problems, including depression, trauma, anxiety disorders, thoughts of revenge, anti-social behaviors, frustration, shame, guilt, phobias [54], dropping out of school [55], loss of trust, loss of safety, poor parental affection, drug abuse, breaking laws and weakness to attend some of the policies or communities organized by the local, sexual reproductive health problems (such as unwanted pregnancies), problems in marital relationships, and domestic violence [56]. We found that without treatment, the severity of the symptoms increased, becoming even more acute for the children of genocide perpetrators.

Results also showed that youth were able to alleviate trauma by finding a space to share their painful memories and emotions. Through this intervention, participating Rwandan youth were able to move forward and forgive their abusers and their descendants. The transmission of trauma and its related effects are gradually gaining the interest of the peace-building community, key among them sociotherapy interventions such as the one discussed here. These are supported by previous studies that showed that no one, regardless of age, is immune to the effects of the trauma caused by our horrific history [31]. Sociotherapy effectively contributed to the promotion of PWB by reducing participants’ mood disturbances and mental disorders, such as depression, hopelessness, anxiety, anger, and stigma. It also helped youth improve their satisfaction with life as well as global life satisfaction. Youth also developed strong relationships with others from their community by sharing dialogues and life experiences. The testimonies and outcomes revealed that sociotherapy contributed to restoration of the hedonic and eudaimonic responses of descendants of both genocide perpetrators and survivors. It was revealed that being with others in the group sessions and feeling support from the group participants helped the youths to become more resilient, make their own choices, and change their lives for the better. These results are supported by previous studies that showed sociotherapy is an effective approach, contributing to improved social cohesion, beneficial changes in participants’ lives, and new life orientation [57].

Sociotherapy, as the community health intervention, was found to be effective in treating the mood disorders and anxiety associated with past traumatic events and transgenerational trauma. The intervention also helped youth to disengage from harmful behaviors and emotional problems arising from their parents’ traumatic histories. Youth who attended the therapeutic sessions, as well as their families and communities, showed they became resilient and gained an increased social engagement. Additionally, youth who attended the sessions became sources of reconciliation and regeneration of socio-cultural values, characterized by becoming mediators and increasing harmony between genocide survivors and perpetrators. These results are in line with previous studies [58, 59]. Post-intervention, participants also are making important life decisions, such as returning to school, creating small businesses, preventing sexual reproductive health issues and their effects, attending governmental policies like (purchasing?) health insurance, and educating their parents on how to shield them from harm. These descendants of individuals convicted of genocide crimes were provided with social healing and individual restoration, instilling in them positive values. These results concur with the findings of prior studies that indicated that the youth of genocide perpetrator developed the spiritual resilience, education, health, and safety [60].

The 1994 Rwandan genocide against the Tutsi exposed an entire population to unimaginable acts of violence and created severe and lasting psychological effects for all involved. The results of this study confirmed that youth recruited to participate in therapeutic sessions developed strategies to cope with life stressors. Their mental well-being was effectively and healthfully reconstructed. These results were in line with previous studies, and also suggested that youth born to genocide perpetrators and survivors healed their wounded hearts and learned to cope with thoughts of revenge, disorganized behaviors characterized by sexual reproductive health problems, shame, and use of drugs and alcohol. Through the sociotherapeutic community health intervention, participants developed social cohesion and appropriate peer relationships. Methods such as sharing their pain with others, joining community support groups, and seeking forgiveness from their victims appeared to elevate these negative effects. Currently, though there are many programs designed to provide counseling services to perpetrators, Rwandans are reluctant to use these resources due to stigma surrounding perpetrators and their descendants seeking therapy. These results were similar to those found in previous studies that indicated that sociotherapy reflected the inner thoughts of the participants, and was effective in reconstructing families and communities [57]. Our results showed that sociotherapy contributed to the reduction of genocide ideologies among youth. They also indicated that youth attending sociotherapy sessions developed feelings of safety, trust, caring, and respect that had been lost due to the indirect and direct effects of genocide. Youth also contributed to the reconciliation between perpetrators and survivors, and to restitution for properties destroyed during genocide.

During the sociotherapy program, youth confirmed that the sessions were the medicine that rebuilt resilience among youth, their families, and communities. Resilience as the fundamental factor for PWB helped youth to make decisions to empower themselves and alter the course of their lives. These findings were in line with previous studies that indicated resilience as the key for PWB in individuals [6]. Indeed, the results confirmed the role of resilience in achieving hedonic and eudemonic well-being in youth, helping them to become autonomous and increase their capacity for living harmoniously with others. In this group therapy program inspired by transactional analysis, youth participants advanced at a fairly rapid pace, with the group itself constituting a therapeutic tool at the service of each member. Sociotherapy contributed to their lives by improving their outlook and allowing for increased control of their feelings, thoughts, behaviors, and relationships. These results were similar to previous studies [57]. This approach is similar to other community-based health interventions (CBHI), including mental health programs, identified as effective in rebuilding the community [61, 62].

After the sessions, youth participants reported that they gradually began to stand up to their aggressive neighbors. Through these interventional sessions, they developed inner psychological healing and rehumanisation that help them to decide not to harass others or heal the psychosocial wounds associated by being harassed due to the harmful histories that affected they parents and cope with risky and disorganized behaviors such as alcohol and drug abuse and sexual and reproductive health problems. Participants also saw improved relationships and social connections, which had been difficult before the program. Because the program was based on individual group members meeting to help heal each other and share both good and harmful experiences, this sociocentric approach differed from an individual-centered approach. In this model, sociotherapy refers to a community-based health intervention that contributes to the psychological well-being of the participants and their communities.

### Strengths and limitations of the study

The current research has numerous prominent strengths. The sociotherapists used validated and standardized tools for anonymously gathering demographic information about the participants. However, several limitations also warrant discussion. Thus, it is recommended that future researchers conduct an experimental study design for enriching the analysis and illuminating the findings of the qualitative study design.

## Conclusion

In conclusion, sociotherapy is an effective community-based health intervention for the promotion of PWB, characterized by hedonic and eudaimonic well-being. This kind of group therapy engaged youth to reflect on the effects of past experiences on their psychological health, sharing their state of mind before sociotherapy, and expressing how the program contributed to their psychological health. Sociotherapy helped the participating youth to grow and develop psychologically by coping with guilt and shame, and improving self-acceptance of their own mistakes and imperfections. They also improved their decisionmaking about new challenges such as returning to school, creating activities for socio-economic welfare, and becoming more social human beings. Due to the sociotherapeutic practices learned in the intervention, youth changed their abnormal behaviors, irrational thinking, and negative emotions for the better. As this interventional approach promoted social cohesion, peace-making, family reconstruction, and effective reintegration of the youth participants’ parents, participants were able to help repair the relationships between descendants of genocide perpetrators and survivors, as well as helping reconcile the genocide perpetrators and survivors themselves.

The findings of this study support providing youth with opportunities to share their experiences. In particular sociotherapy is recommended because it is an effective method for reducing the transmission of trauma and reconciling genocide perpetrators and survivors. Researchers recommend further research is conducted on: (1) exploration of the applicability of the stages of behavioral changes amongst youth participants in sociotherapy, (2) the barriers to the effectiveness of sociotherapy on the psychological well-being of youth. An experimental study design is recommended for assessing the efficacy of such a program.

## Data Availability

The datasets generated and analyzed during the current study are available from the corresponding author upon reasonable request. These data are also available at the Prison Fellowship Rwanda (PFR), where the main author works in the program of sociotherapy.

## Abbreviations

CBHI: Community-Based Health Intervention
EKN: Embassy of the Kingdom of the Netherlands
PWB: Psychological Well-being
PFR: Prison Fellowship Rwanda
CBS: Community-Based Sociotherapy
E.A.R: Eglise Anglicaine du Rwanda (Anglican Church of Rwanda)
